# Identification of a TNF-α inducer MIC3 originating from the microneme of non-cystogenic, virulent *Toxoplasma gondii*

**DOI:** 10.1038/srep39407

**Published:** 2016-12-21

**Authors:** Jingfan Qiu, Lijuan Wang, Rong Zhang, Ke Ge, Hongfei Guo, Xinjian Liu, Jinfeng Liu, Delong Kong, Yong Wang

**Affiliations:** 1 Department of Pathogen Biology, Key Laboratory of Pathogen Biology of Jiangsu Province, Nanjing Medical University, Nanjing, 211166, China

## Abstract

*Toxoplasma gondii* is an opportunistic parasite with avirulent cystogenic and highly virulent non-cystogenic isolates. Although non-cystogenic strains are considered the most virulent, there are also marked genetic and virulence differences among these strains. Excretory-secretory antigens (ESAs) of *T. gondii* are critical for the invasion process and the immune response of the host. To better understand the differences in virulence between non-cystogenic *T. gondii* isolates, we studied ESAs of the RH strain (Type I), and the very prevalent in China, but less virulent TgCtwh3 strain (Chinese 1). ESAs of RH and TgCtwh3 triggered different levels of TNF-α production and macrophage M1 polarization. Using iTRAQ analysis, 27 differentially expressed proteins originating from secretory organelles and surface were quantified. Of these proteins, 11 microneme-associated proteins (MICs), 6 rhoptry proteins, 2 dense granule proteins and 5 surface proteins were more abundant in RH than in TgCtwh3. The protein-protein correlation network was employed to identify the important functional node protein MIC3, which was upregulated 5-fold in RH compared with TgCtwh3. MIC3 was experimentally confirmed to evoke a TNF-α secretory response, and it also induced macrophage M1 polarization. This result suggests that MIC3 is a potentially useful immunomodulator that induces TNF-α secretion and macrophage M1 polarization.

*Toxoplasma gondii* is a successful protozoan parasite that is prevalent worldwide in humans and in many warm-blooded animals. Most infections caused by *T. gondii* are asymptomatic in humans, but infection acquired during pregnancy may lead to severe congenital toxoplasmosis, and immunosuppressed individuals, including AIDS patients, may suffer severe diseases, including toxoplasmic encephalitis^1^. In addition to the immune status of the host, the virulence of *T. gondii* strains also influences clinical outcomes. Cystogenic *T. gondii* strains form chronic tissue cysts and are usually avirulent. Non-cystogenic isolates tend to be highly virulent in mice, making mice good research models for acute toxoplasmosis.

Non-cystogenic *T. gondii* strains vary genetically and in terms of virulence. The RH strain (Type I genotype) is a common virulent isolate in North America and Europe, and strain TgCtwh3 (Chinese 1 genotype) is common in China^2^,^3^. Previous studies have demonstrated that strain TgCtwh3 is less virulent than strain RH. For example, Li *et al*.^3^ found that the average number of survival days post-infection of mice with 10 tachyzoites was 9 days for strain RH and 12 days for strain TgCtwh3. The dose-dependent hazard ratio for mice infected with RH tachyzoites was more than 3-fold higher than that with TgCtwh3 tachyzoites^3^. However, the underlying molecular reasons for this discrepancy are unclear.

Proteomic techniques offer an excellent platform to investigate *T. gondii*. Proteomic studies in *T. gondii* initially focused on the identification of proteins in the total cell extract^4^,^5^,^6^. Modern quantitative proteomics has mainly focused on North American and European *T. gondii* isolates^7^,^8^,^9^. However, approximately 7.9% of the human population^10^ and 18.0% of retail pork^11^ in China tested positive for anti-*T. gondii* antibodies. The prevalent *T. gondii* isolates in China differ from the American and European isolates. Zhou *et al*.^12^ explored the complete proteomic profile of *T. gondii* tachyzoites of the Chinese 1 TgC7 strain in comparison to Western isolates and identified several differentially abundant proteins using 2D difference gel electrophoresis (DIGE) combined with matrix-assisted laser desorption/ionization time-of-flight mass spectrometry (MALDI-TOF MS). Unfortunately, although there is much data on the total extract of tachyzoites, the specific proteins that are important for *T. gondii* pathogenicity remains to be determined.

Excretory-secretory antigens (ESAs) produced by *T. gondii* tachyzoites represent the majority of the circulating antigens in serum and cerebrospinal fluid from hosts with acute infection^13^,^14^,^15^. They are among the first targets of the host cellular and humoral immune response, which limits the infection^13^,^14^,^15^,^16^,^17^,^18^. Most ESAs are secreted from the *T. gondii* apical organelle complex, consisting of rhoptries, micronemes and dense granules^19^,^20^,^21^. The rhoptry and microneme ESAs promote adherence and host cell invasion^19^,^21^. The dense granule proteins are important for the formation of the parasitophorous vacuole (PV), which shields the pathogen from the nitric oxide produced by M1-polarized macrophages for defence against the parasite^19^. Thus, *T. gondii* ESAs are important antigens and also play an important role in the invasion process. Hence, targeted studies of ESAs should be conducted to reveal important proteins associated with virulence.

The defensive host reaction against virulent *T. gondii* involves TNF-α production, and M1 polarization of macrophages, including the induction of the iNOS and IL-6 genes to produce nitric oxide and IL-6, respectively^22^,^23^,^24^. In this study, we reported that the ESAs from the less virulent Chinese 1 TgCtwh3 strain induced lower TNF-α production, and a fewer number of M1 macrophages than the *T. gondii* Type I RH (Western) strain. We employed isobaric Tags for Relative and Absolute Quantification (iTRAQ) for comparative proteome analysis to identify differentially expressed ESA proteins in the two non-cystogenic, virulent *T. gondii* strains. Twenty-seven differentially abundant ESA proteins were identified, and five of them were shown to be transcriptionally induced using qPCR. The soluble adhesin, MIC3^19^,^25^, from the microneme, was 5-fold more abundant in strain RH than in strain TgCtwh3. Pure MIC3 induced TNF-α production and macrophage M1-dominant polarization. Therefore, MIC3 played a very important role in *T. gondii* virulence. These results highlight additional directions of study to better understand MICs.

## Results

### Tachyzoites and ESAs of the Chinese 1 TgCtwh3 strain induced lower levels of TNF-α production than those of the Type I RH strain

**Figure 1:**
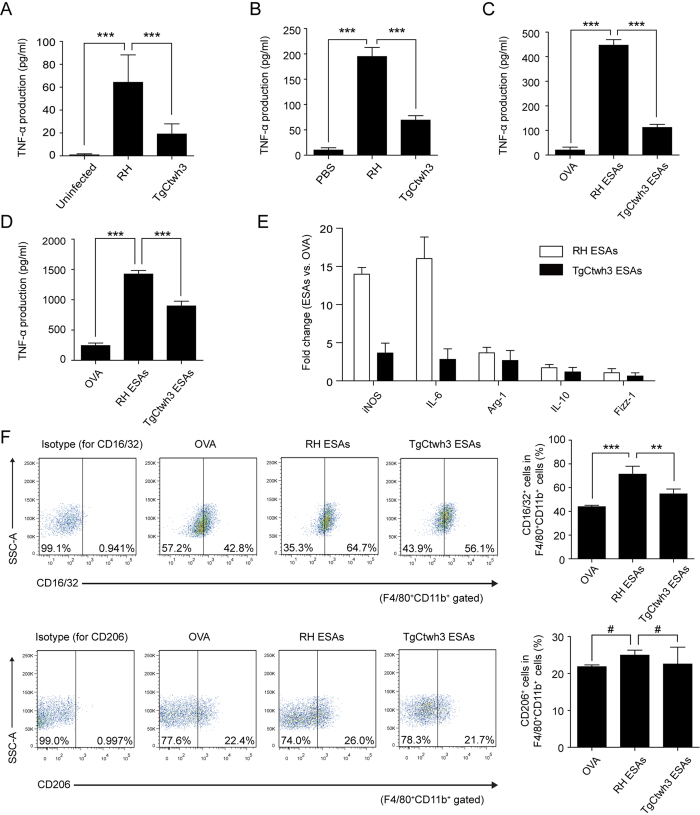
*T. gondii* infection and ESA treatment induced TNF-α secretion in C57BL/6 mice. (**A**) Serum levels of TNF-α in mice injected with *T. gondii* RH and TgCtwh3 tachyzoites. Each bar indicates the mean value ± S.D. (n = 6). Significance was analysed using one-way ANOVA. ***P < 0.001. (**B**) Freshly isolated mouse splenocytes were infected with *T. gondii* RH and TgCtwh3 tachyzoites for 72 h. Each bar indicates the mean value ± S.D. (n = 6). Significance was analysed using one-way ANOVA. ***P < 0.001. (**C,D**) Freshly isolated mouse splenocytes (**C**) and peritoneal macrophages (**D**) were stimulated with 10 μg/ml *T. gondii* RH ESAs and *T. gondii* TgCtwh3 ESAs for 72 h. TNF-α in the supernatants was quantified by ELISA. Data are represented as the means ± S.D. (n = 6). Significance was analysed using one-way ANOVA. ***P < 0.001. (**E**) Peritoneal macrophages treated with OVA, RH ESAs and TgCtwh3 ESAs were analysed quantitatively using qPCR for the indicated M1 (iNOS, IL-6) or M2 (Arg-1, IL-10, Fizz-1) marker genes. Data are presented as the fold change relative to the control (OVA-treated group). GAPDH was used as an internal control. 1 = no change. (**F**) Peritoneal macrophages were stimulated with 10 μg/ml OVA, RH ESAs and TgCtwh3 ESAs for 72 h. The expression levels of CD16/32 (M1) and CD206 (M2) were evaluated by FCM analysis. One representative of 3 independent experiments is shown. Data are expressed as the means ± S.D. (n = 3). Significance was analysed using one-way ANOVA. ***P < 0.001, **P < 0.01, ^#^*P* > 0.05.

The virulence of the RH and TgCtwh3 strains has been studied by measuring the survival times of mice inoculated with *T. gondii* tachyzoites^3^. However, differences in the host immune responses to these strains have not yet been evaluated. We wanted to elucidate why Chinese 1 TgCtwh3 appeared to be less virulent than the Type I RH strain. Previous studies have demonstrated that TNF-α production is involved in the pathogenesis of toxoplasmosis^22^. TNF-α reduces *T. gondii* replication both *in vivo* and *in vitro*, but it also causes inflammation^26^. To compare the level of TNF-α induced after infection with *T. gondii* strains RH and TgCtwh3, 1 × 10^6^ freshly harvested *T. gondii* RH or TgCtwh3 tachyzoites, or phosphate buffered saline (PBS) control were intraperitoneally injected into C57BL/6 mice. After 3 days, the levels of TNF-α in mouse serum were 64.24 ± 23.99, 19.08 ± 8.90, and 0.82 ± 0.94 pg/ml for RH, TgCtwh3, and the control, respectively (Fig. 1A). The ratio of TNF-α produced in response to RH and TgCtwh3 was approximately 3-fold, and RH produced significantly more TNF-α than TgCtwh3 (*P* < 0.001). Therefore, the reported *T. gondii* dose-dependent hazard ratio^3^ and the level of TNF-α production seemed to be correlated (*r* > 0.6, *P* < 0.05). In addition, we assessed the parasite burden in the spleens of infected mice. On day 3 post-inoculation, the splenic parasite burden was similar in mice that were injected with RH or TgCtwh3 tachyzoites (*P* > 0.05, Supplementary Fig. S1). This result suggested that the difference in TNF-α production *in vivo* was not attributed to the growth and replication discrepancy of *T. gondii*.

To determine whether *T. gondii* RH tachyzoites can induce higher levels of TNF-α not only *in vivo* but also *in vitro*, 1 × 10^6^ freshly harvested *T. gondii* RH or TgCtwh3 tachyzoites, or PBS controls were added to splenocytes of C57BL/6 mice. After 3 days, the TNF-α levels were 194.84 ± 17.98, 69.37 ± 8.56, and 10.12 ± 4.58 pg/ml for RH, TgCtwh3, and the control, respectively (Fig. 1B). The ratio of TNF-α produced (RH: TgCtwh3) was approximately 3:1, and greater TNF-α production was induced by RH tachyzoites than TgCtwh3 tachyzoites *in vitro* (*P* < 0.001).

*T. gondii* is known to secrete many proteins that are collectively known as ESAs. Some of these proteins are important antigens or are known to be involved in the infection process^15^. To investigate whether *T. gondii* ESAs contribute to the discrepancy in TNF-α secretion induced by RH and TgCtwh3 tachyzoites, freshly isolated mouse splenocytes were stimulated with 10 μg/ml RH, TgCtwh3 ESAs (free of endotoxin), or OVA (ovalbumin) control for 3 days, and the TNF-α levels were 446.52 ± 22.86, 112.32 ± 12.67, and 20.36 ± 11.96 pg/ml, respectively (Fig. 1C). RH ESAs induced significantly higher amounts of TNF-α than TgCtwh3 ESAs (*P* < 0.001). The ratio of TNF-α produced (RH-OVA: TgCtwh3-OVA) was approximately 4:1. Therefore, the differential induction of TNF-α production was similar for mice infected with live *T. gondii* tachyzoites.

Macrophages are known to secrete TNF-α. We wanted to determine whether the *T. gondii* ESA preparations could stimulate TNF-α production *in vitro* in macrophages. Freshly isolated mouse peritoneal macrophages were stimulated with 10 μg/ml RH, TgCtwh3 ESAs, or OVA for 3 days. For peritoneal macrophages, TNF-α production was 1424.46 ± 60.15, 898.28 ± 78.59, and 245.00 ± 41.45 pg/ml for RH ESAs, TgCtwh3 ESAs and OVA, respectively (Fig. 1D). RH ESAs induced significantly more TNF-α than TgCtwh3 ESAs (*P* < 0.001). The ratio of TNF-α produced (RH-OVA: TgCtwh3-OVA) was approximately 2:1, showing a correlation between the reported *T. gondii* hazard ratio^3^ and ESA-induced TNF-α secretion (*r* > 0.6, *P* < 0.05). Thus, we decided to focus on *T. gondii* ESAs to identify the one(s) responsible for the induction of TNF-α secretion in C57BL/6 mice.

Macrophages are necessary for host protection during *T. gondii* infection^27^. Acute *T. gondii* infection initiates M1-dominant polarization of macrophages, which produce high levels of NO and proinflammatory cytokines such as TNF-α and IL-6^22^,^23^,^28^,^29^. M1 macrophages destroy intracellular pathogens by releasing NO, which is known to be a major effector molecule in macrophage-mediated cytotoxicity^27^. However, an over-production of NO may result in serious immunopathology. In contrast to M1-polarized macrophages, M2-polarized macrophages rarely express inducible nitric oxide synthase (iNOS) but express high levels of arginase 1 (Arg-1), which skews the metabolic pathway of NO to produce proline^27^. Other genes that are induced by M2 macrophages include IL-10 and Fizz-1 (also called RELM-α)^27^. To characterize the differentiation of macrophage polarization, qPCR was used to measure the amounts of the above marker genes in peritoneal macrophages 3 days after stimulation with 10 μg/m *T. gondii* ESAs. Compared with the OVA control, RH ESAs induced iNOS and IL-6 mRNA upregulation more than 13-fold, but Arg-1, IL-10 and Fizz-1 did so less than 4-fold (Fig. 1E). This result suggested that macrophages could change their weak polarization state to a profoundly M1-dominant polarized state in response to RH ESA stimulation. TgCtwh3 ESAs were less effective at inducing iNOS and IL-6. The mRNA for these genes and for Arg-1, IL-10 and Fizz-1 was upregulated less than 4-fold. Thus, *T. gondii* TgCtwh3 ESAs were less effective than RH ESAs in stimulating TNF-α production and macrophage M1 polarization.

We also analysed the expression of selected cell surface markers, CD16/32, CD206, F4/80 and CD11b, using flow cytometry (FCM), to characterize peritoneal macrophage polarization (Fig. 1F). The cells expressing CD16/32^+^F4/80^+^CD11b^+^ or CD206^+^F4/80^+^CD11b^+^ were defined as M1 or M2 macrophages, respectively. M1 macrophages were the predominant macrophages in both the RH ESA group and the TgCtwh3 ESA group. Compared with the OVA control, the percentage of M1 macrophages significantly increased after stimulation with RH ESAs (*P* < 0.001). TgCtwh3 ESAs were less effective than RH ESAs at inducing CD16/32 expression (*P* < 0.01). However, the percentage of M2 macrophages did not show any significant differences in OVA and ESA groups. This result further indicated that RH ESAs were more effective than TgCtwh3 ESAs at inducing M1-dominant polarized state.

### Overview of the proteomic analysis of ESA proteins

A quantitative iTRAQ-LC-MS/MS proteomics approach was employed herein to assess differences between the amounts of ESA proteins produced by strains RH and TgCtwh3 (Supplementary Fig. S2). Two independent cultivations of both the RH and TgCtwh3 strain were used, resulting in two biological replicates for each strain.

**Figure 2:**
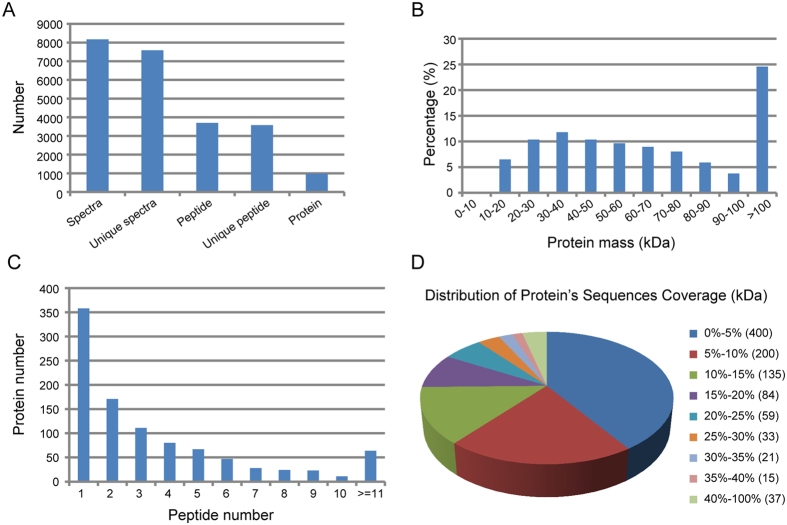
Summary of iTRAQ quantitative protein analysis of the *T. gondii* ESA proteome. (**A**) Numbers of identified spectra, unique spectra, peptides, unique peptides, and proteins. (**B**) Mass distribution of identified *T. gondii* proteins. (**C**) Numbers of identified peptides per *T. gondii* protein. (**D**) Sequence coverage (number of *T. gondii* proteins).

The raw data were filtered to eliminate low-scoring spectra, and the qualified spectra were matched to 984 *T. gondii* proteins (Fig. 2A). A total of 301,033 spectra were obtained. We identified 8,168 spectra, among which 7,579 were unique. Analytical results using the Mascot 2.3.02 search engine resulted in the acquisition of 3,699 *T. gondii* peptides, which included 3,578 unique peptides representing 984 *T. gondii* proteins. The protein mass distribution >10 kDa followed a normal distribution (Fig. 2B) as follows: 0–50 kDa, 39.1%; 50–100 kDa, 36.3%; and >100 kDa, 24.6%. Three hundred fifty-eight proteins were represented by only a single peptide, which was not considered sufficient for the reliable identification of a protein, and these proteins were not used in further analyses. Six hundred twenty-six ESA proteins that were represented by two or more peptides, were further assessed (Fig. 2C). The distribution of protein sequence coverage is shown in Fig. 2D. Protein sequence coverage representing less than 10%, 10–20%, 20–30%, 30–40%, and 40–100% variation accounted for 60.97%, 22.26%, 9.35%, 3.66%, and 3.76% coverage, respectively.

The reproducibility of the quantitative proteomic analyses was assessed by comparing the two biological replicates from each strain. The difference was plotted versus the percentage of proteins identified. The results showed that 80% of the proteins had a delta error difference of less than 0.15–0.25, and more than 95% of the proteins had a delta error difference of less than 0.5, suggesting that the biological noise was reasonably low (Supplementary Fig. S3).

### Functional categories of differentially expressed proteins

**Figure 3:**
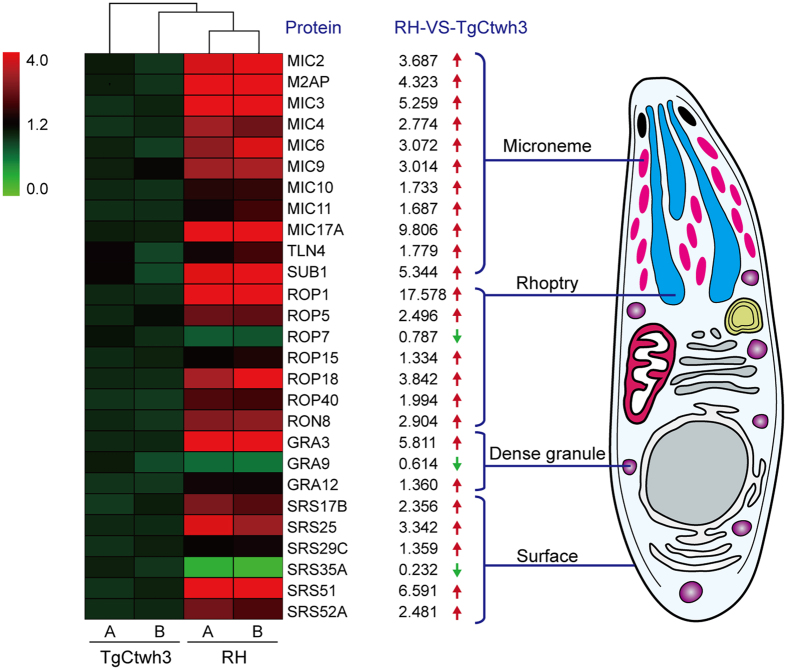
Differential expression of secretory organelle proteins (microneme, rhoptry, and dense granule) and surface proteins (SRSs). The heat map was generated using MultiExperimentViewer (version 4.8.1) to visualize differences in protein expression between RH ESAs and TgCtwh3 ESAs. A and B in the heat map indicate two biological replicates of each strain. The schematic diagram of *T. gondii* shows the subcellular localization of the proteins listed in the heat map.

Greater than 1.2-fold differences between the amounts of proteins observed in the ESAs of *T. gondii* strains RH and TgCtwh3 were considered significant (*P* < 0.05). Smaller differences were ignored. The iTRAQ analytical results revealed 48 proteins that were more abundant and 18 proteins that were less abundant in the ESAs of *T. gondii* strain RH than in strain TgCtwh3. Detailed information is provided for each protein in Supplementary Table S1. Twenty-one of the 66 differentially abundant ESA proteins originated from *T. gondii* secretory organelles (microneme, rhoptry, and dense granule), and 6 were from the *T. gondii* cell surface (Table 1, Fig. 3). The vast majority of the 27 known secretory or surface proteins were highly expressed in the RH strain, including 9 microneme proteins (MICs), 6 rhoptry proteins (ROPs), 2 dense granule proteins (GRAs), 5 surface proteins (SRSs), and 2 proteases (toxolysin 4 and subtilisin 1). In contrast, only 1 ROP, 1 GRA, and 1 SRS showed elevated expression in TgCtwh3 ESAs compared with RH strain ESAs. SRSs appeared in ESAs, likely due to the proteolytic shedding from the parasite^20^,^30^. Toxolysin 4 (TLN4) is a putative metalloprotease that is processed into multiple proteolytic fragments within the *T. gondii* secretory system^31^. Protease subtilisin 1 (SUB1) is essential for cell surface processing of micronemal adhesive protein complexes and efficient adhesion of tachyzoites^32^. Several proteins from non-secretory organelles also appeared in the ESAs (Supplementary Table S1) and were likely released from dead tachyzoites.

### Gene Ontology (GO) annotation

GO annotation (http://www.geneontology.org/) was performed to better understand the biological functions of the 66 differentially expressed *T. gondii* ESA proteins. The proteins were classified based on biological processes, cellular components, and molecular function according to the GO database. Information from the GO database suggested that some of the 66 differentially expressed *T. gondii* ESA proteins may be important for virulence, including attachment to, and invasion of target cells.

**Figure 4:**
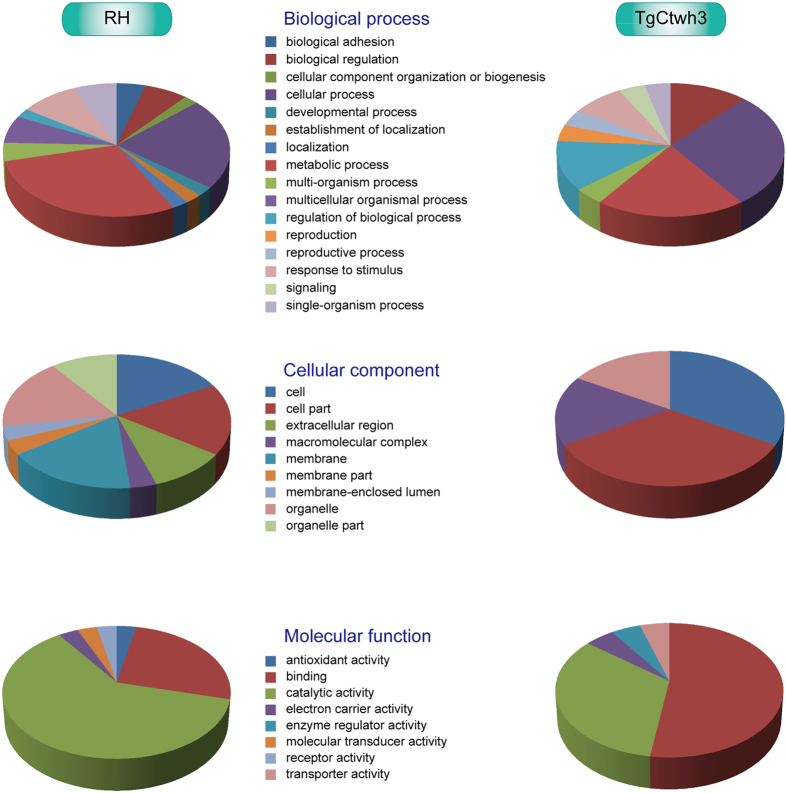
Summary of GO functional predictions of upregulated *T. gondii* ESA proteins in RH or TgCtwh3. Upregulated proteins were defined as those that showed greater than a 1.2-fold change in relative abundance and a *P* value < 0.05. There are 48 upregulated proteins in RH and 18 upregulated proteins in TgCtwh3.

Enrichment analyses of GO annotations were performed on proteins that were upregulated in *T. gondii* RH ESAs or TgCtwh3 ESAs. Functions involved in important biological processes, such as metabolic processes, cellular processes, and biological regulation, were significantly overrepresented in each *T. gondii* ESAs (Fig. 4). The majority of the highly expressed proteins in RH ESAs or TgCtwh3 ESAs originated from organelles or macromolecular complexes. However, upregulated proteins in the RH strain contained more membrane and extracellular proteins than the TgCtwh3 strain. Moreover, these differentially expressed proteins were associated with important molecular functions. Functions involved in binding and catalytic activity were found to be significantly overrepresented in each *T. gondii* strain. However, several functions, such as antioxidant activity, molecular transducer activity, and receptor activity, were significantly underrepresented in *T. gondii* TgCtwh3.

### Protein-protein correlation of differentially expressed proteins in *T. gondii* ESAs

**Figure 5:**
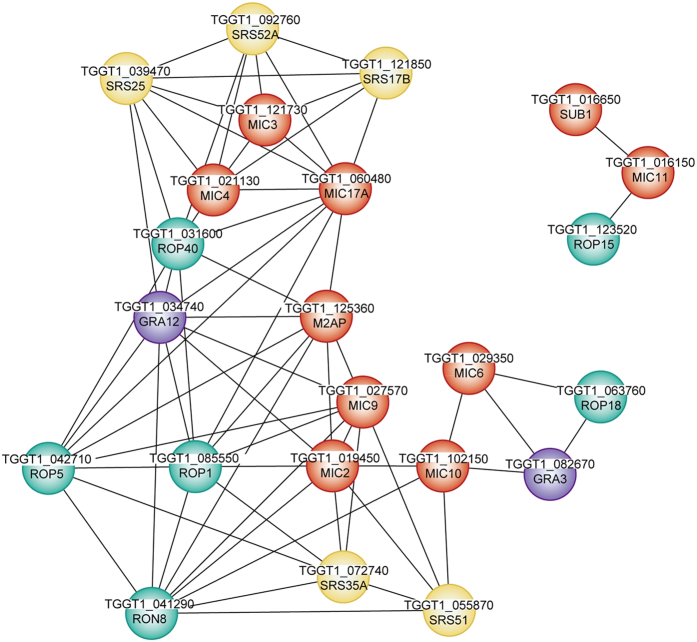
Correlation between differentially expressed proteins in *T. gondii* RH ESAs and TgCtwh3 ESAs. Protein-protein correlation based on a correlation analysis indicates possible interactions between certain proteins. The line between two balls represents the Pearson’s correlation coefficient (*r*) for these two proteins > 0.6 or < −0.6, *P* < 0.01. Red, microneme-associated proteins; green, rhoptry proteins; purple, dense granule proteins; yellow, surface proteins.

The exploration of protein-protein correlations is a new strategy to identify important functional nodes controlling crucial networks for parasite invasion and survival. A correlation analysis was conducted based on the Pearson’s correlation coefficient to select key functional nodes among several upregulated and downregulated proteins in *T. gondii* RH ESAs. Clear correlations (*r* > 0.6 or < −0.6, *P* < 0.01) could be visualized between MICs, ROPs, GRAs, or SRSs by Cytoscape^33^. A total of 17 secretory proteins were highly correlated with other proteins, including 7 MICs, 4 ROPs, 5 SRSs, and GRA12 (Fig. 5). This finding was indicative of the ability of these proteins to form complexes, or to interact during the attachment or invasion process of *T. gondii*. Among these proteins, MIC3 was an important functional node protein and was subsequently characterized.

### Transcriptional analysis of five differentially expressed *T. gondii* ESA proteins

**Figure 6:**
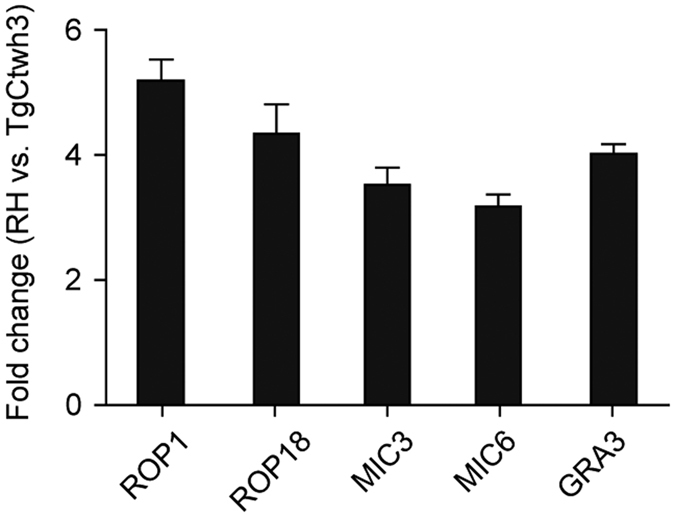
Relative transcription levels of five highly expressed proteins in the RH compared with the TgCtwh3 strain. The samples were tested by quantitative RT-PCR using gene-specific primers (Supplementary Table S2), and GAPDH served as an internal control. Data are expressed as the means ± S.D. (n = 6). 1 = no change.

Rop1, Rop18, MIC3, MIC6 and GRA3 were selected for transcriptional analysis because they were >3-fold more abundant in the ESAs of strain RH than in strain TgCtwh3 and highly correlated with other proteins. Quantitative RT-PCR using gene-specific primers (Supplementary Table S2) showed that the mRNA of all five genes was >3-fold more abundant in strain RH than in TgCtwh3 (Fig. 6). This finding was consistent with the observed protein levels, suggesting that the corresponding genes were under transcriptional control.

### Recombinant *T. gondii* MIC3 induces TNF-α secretion in macrophages

**Figure 7:**
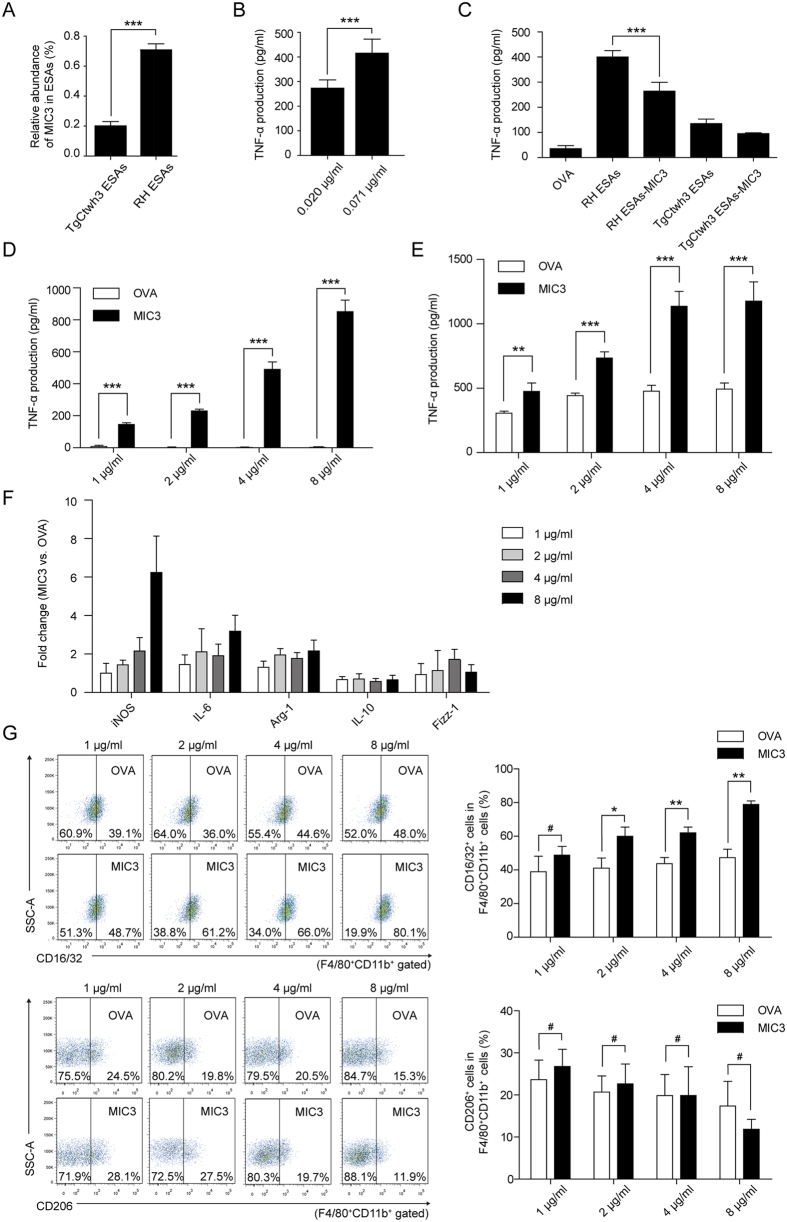
Effects of *T. gondii* MIC3 on TNF-α secretion in mice. (**A**) Abundance of MIC3 in RH ESAs and TgCtwh3 ESAs detected by ELISA. Data are presented as the means ± S.D. (n = 6). Significance was analysed using the two-tailed Student’s *t*-test. ***P < 0.001. (**B**) TNF-α secretion levels by peritoneal macrophages stimulated separately with 0.020 μg/ml and 0.071 μg/ml MIC3 (abundance of MIC3 in 10 μg/ml TgCtwh3 and RH ESAs, Fig. 7A). Each bar indicates the mean value ± S.D. (n = 6). Significance was analysed using the two-tailed Student’s *t*-test. (**C**) The levels of TNF-α secretion by mouse splenocytes stimulated with 10 μg/ml OVA, RH ESAs, MIC3-depleted RH ESAs, TgCtwh3 ESAs and MIC3-depleted TgCtwh3 ESAs. Data are represented as the means ± S.D. (n = 6). Significance was analysed using one-way ANOVA. (**D,E**) The levels of TNF-α secretion by mouse splenocytes (**D**) and peritoneal macrophages (**E**) stimulated with different concentrations of MIC3 (1, 2, 4 and 8 μg/ml). Data are represented as the means ± S.D. (n = 6). Significance was analysed using the two-tailed Student’s *t*-test. **P < 0.01. (**F**) Peritoneal macrophages treated with different concentrations of MIC3 and OVA (1, 2, 4 and 8 μg/ml) were analysed by qPCR to evaluate the mRNA expression of iNOS, IL-6, Arg-1, IL-10 and Fizz-1, which are marker genes for M1 and M2 polarization. GAPDH served as an internal control. Bars represent the relative mRNA levels of iNOS, IL-6, Arg-1, IL-10 and Fizz-1 produced by macrophages stimulated with MIC3 compared with macrophages stimulated with the same concentration of OVA. Error bars represent the S.D. from data pooled from six independent experiments. 1 = no change. (**G**) Peritoneal macrophages were stimulated with different concentrations of MIC3 and OVA (1, 2, 4 and 8 μg/ml) for 72 h. The expression levels of CD16/32 (M1) and CD206 (M2) were evaluated by FCM analysis. Data are presented as the means ± S.D. (n = 3). Significance was analysed using the two-tailed Student’s *t*-test. *P < 0.05, ^#^*P* > 0.05.

In our study, MIC3 was found to be an important functional node protein and >3-fold upregulated in RH compared with TgCtwh3 both at the protein and transcriptional levels. Previous results demonstrated that MIC3 could bind to the surface of host cells and aid initial parasite invasion^34^, but little is known about whether MIC3 is responsible for TNF-α production. Our aim was to evaluate whether pure *T. gondii* MIC3 protein was an important inducer of TNF-α secretion by mouse peritoneal macrophages. First, we determined the sequences of MIC3 from *T. gondii* RH and TgCtwh3 by sequencing. The amino acid sequences of MIC3 from *T. gondii* RH and TgCtwh3 are identical (Supplementary Fig. S4). To generate biologically meaningful results, we wanted to use the same amount of recombinant MIC3 as was present in the ESA preparations. iTRAQ proteomics measures only relative levels of proteins. The absolute amounts of MIC3 were measured using an enzyme linked immunosorbent assay (ELISA) with anti-MIC3 antibodies. The ESA preparations contained 10 μg/ml proteins. The ESA preparations from strains RH and TgCtwh3 contained 0.071 and 0.020 μg/ml MIC3 protein, respectively (Fig. 7A). Three-day treatment of mouse peritoneal macrophages with 0.071 or 0.020 μg/ml recombinant MIC3 resulted in the secretion of 415.71 ± 57.18 or 273.35 ± 33.40 pg/ml TNF-α, respectively (Fig. 7B). The ratio between the two values was approximately 1.5, as observed for the RH and TgCtwh3 ESA preparations in peritoneal macrophages (Fig. 1D). However, the amounts of secreted TNF-α were approximately only 30% of those obtained by treatment with ESA preparations.

To establish whether MIC3 is the determining factor in the induction of TNF-α, we performed immunoprecipitation (IP) to deplete MIC3 from ESAs (Fig. 7C). The results suggested that MIC3-depleted RH ESAs induced a much lower level of TNF-α (approximately 2/3) than RH ESAs (*P* < 0.001). These findings demonstrate that MIC3 is a dominant factor in the induction of TNF-α.

We measured the level of TNF-α secretion by splenocytes stimulated with different concentrations of MIC3 to further evaluate the role of MIC3 in this process. The levels of TNF-α in the experimental groups with MIC3 were significantly higher than those in OVA controls at 1 (145.82 ± 10.05 pg/ml vs. 7.88 ± 7.15 pg/ml, *P* < 0.001), 2 (230.66 ± 10.47 pg/ml vs. 0.14 ± 5.57 pg/ml, *P* < 0.001), 4 (490.05 ± 46.17 pg/ml vs. 1.43 ± 2.79 pg/ml, *P* < 0.001), and 8 μg/ml (850.99 ± 71.79 pg/ml vs. 1.92 ± 4.42 pg/ml, *P* < 0.001) (Fig. 7D). This result indicated that MIC3 was an effective inducer of TNF-α. During acute infection with *T. gondii*, TNF-α acts synergistically with IFN-γ, resulting in the elimination of intracellular pathogens^35^. However, MIC3 did not induce any IFN-γ secretion compared with OVA in splenocytes (Supplementary Fig. S5). We measured the level of TNF-α secretion by macrophages stimulated with MIC3 because TNF-α was first described as a factor secreted by macrophages that can induce intense tumour necrosis. The same result was observed in peritoneal macrophages stimulated with MIC3. Notably, the TNF-α levels were higher in the MIC3 groups than in the OVA controls at 1 (475.54 ± 64.98 pg/ml vs. 306.49 ± 15.27 pg/ml, *P* < 0.01), 2 (734.58 ± 49.13 pg/ml vs. 442.88 ± 18.48 pg/ml, *P* < 0.001), 4 (1136.67 ± 115.12 pg/ml vs. 477.40 ± 45.26 pg/ml, *P* < 0.001), and 8 μg/ml (1176.74 ± 149.07 pg/ml vs. 492.80 ± 47.30 pg/ml, *P* < 0.001) (Fig. 7E). These results showed that MIC3 induced TNF-α secretion in macrophages. In addition, clear morphological changes in peritoneal macrophages were observed after MIC3 stimulation (Supplementary Fig. S6). Round cells became elongated and firmly adherent after MIC3 stimulation. Thus, activated macrophages were dominant in the MIC3 group compared with the OVA group as shown by morphological changes typical of differentiation.

iNOS, IL-6, Arg-1, IL-10 and Fizz-1 mRNA expression levels precisely reflect the overall polarization status. Thus, the mRNA expression levels of the primary M1 and M2 polarization markers, iNOS, IL-6, Arg-1, IL-10 and Fizz-1, were evaluated in macrophages in our study. MIC3 stimulation increased both iNOS and Arg-1 expression compared with OVA (Fig. 7F). The overall polarization of macrophages shifted to M1 dominance after stimulation with 8 μg/ml MIC3 because the expression of iNOS significantly increased (>6-fold) and the expression of Arg-1, Fizz-1 and IL-10 showed no notable change (<2.5-fold). In addition, this increased iNOS expression was accompanied by a slight increase in the expression of IL-6 (>3-fold). Furthermore, we analysed the expression of selected cell surface markers, CD16/32 and CD206, to characterize peritoneal macrophage polarization (Fig. 7G). MIC3 stimulation elevated the percentage of CD16/32^+^ macrophages compared with OVA. In the 8 μg/ml MIC3 group, approximately 80% of the F4/80^+^CD11b^+^ cells were CD16/32^+^ macrophages. These results showed that MIC3 played an important role in the induction of macrophage polarization.

## Discussion

*T. gondii* has attracted attention as a substantial public health risk in China, with a 7.9% seropositive rate in humans^10^. The Chinese 1 lineage is a dominant lineage from swine, cats, and humans in mainland China^2^. Clarifying the virulence discrepancy between Chinese 1 and Type I (very prevalent in North America and Europe) genotypes is essential to gain a better understanding of *T. gondii* isolates in the Chinese 1 genetic background. The *T. gondii* Chinese 1 TgCtwh3 strain is less virulent than the Western Type I RH strain according to mouse hazard ratio tests. We found that in C57BL/6 mice, the *T. gondii* strain TgCtwh3 induced less TNF-α production than strain RH (Fig. 1A). This finding was significant because TNF-α was believed to be an important defence against *T. gondii* infection and a powerful inducer of inflammation^22^,^36^. Special efforts were implemented to remove potential lipopolysaccharides (LPS) from the ESA preparations, which are potent TNF-α inducers. Next, we confirmed that the *T. gondii* ESA preparations induced TNF-α production in mouse splenocytes or peritoneal macrophages, similar to tachyzoites infection (Fig. 1C,D). Therefore, we hypothesized that the differential expression of one or more specific ESA proteins may be responsible for the difference in virulence between the two *T. gondii* strains.

Proteomic information for the Chinese 1 lineage is scarce and insufficient despite recent developments in this field. In the present work, we investigated the proteomic profiles from tachyzoite ESAs of two *T. gondii* lineages belonging to the Chinese 1 and Type I genotypes. The differentially expressed ESA proteins were compared using iTRAQ. A total of 27 proteins were identified, including 9 MICs, 7 ROPs, 3 GRAs, 6 SRSs, and 2 proteases. Among these proteins, Rop1, Rop18, MIC3, MIC6, and GRA3 were further confirmed to be highly expressed (>3-fold) in the RH strain both at the protein and mRNA levels. Several of these proteins, have been directly linked to the infection process by way of functions such as cell adhesion, or formation of the host parasitophorous vacuole (PV) that protects *T. gondii* from host cell defences. For example, ROP1 initially lines the vacuolar space but disappears as the vacuole matures, functioning as a penetration-enhancing factor^19^,^20^. Deletion of ROP18 resulted in complete attenuation of virulence in *T. gondii* South American strains^37^. MIC3 is the adhesive component of the MIC3/8 complex because it has a demonstrable host cell binding property, an NH_2_-terminal chitin binding (CB)-like (CBL) domain^38^. MIC6 of the MIC1/4/6 complex not only functions to anchor the adhesive complex to the membrane of the host cell but also acts as a “quality control” protein that is required to release the complex from a checkpoint located in the Golgi apparatus^39^. GRA3 has been shown to be important for the structure of the PV and nutrient acquisition for the parasite^40^. Previous studies have demonstrated that these five proteins played an important role in virulence. Thus, their upregulated expression in the RH strain contributes to the discrepancy in virulence between the RH and TgCtwh3 strains.

The release of TNF-α, which is a 157 amino acid cytokine, can be triggered by various molecules of microbial origin, of which the most active is bacterial LPS endotoxin^41^,^42^. However, under conditions that exclude any impact of contaminating endotoxin, a number of intracellular parasites, including the malarial parasites, *Leishmania* and *Trypanosoma cruzi*, could also induce the release of TNF-α^43^,^44^,^45^. As an intracellular pathogen, *T. gondii* can also induce TNF-α production^22^. Toxoplasmosis patients, especially those who develop ocular or cerebral toxoplasmosis, produced high levels of TNF-α^22^. In our study, the ESA preparations from *T. gondii* were comparable to those of live tachyzoites in terms of their ability to induce TNF-α. This result suggested that specific antigens from *T. gondii* ESAs might be responsible for TNF-α production.

Stimulation of TNF-α synthesis by *T. gondii* plays an important role in the regulation of the resistance mechanisms and pathologic effects induced by this organism. Nevertheless, little is known about the parasite molecules involved in this process. Previous studies have largely focused on glycosylphosphatidylinositols (GPIs) from *T. gondii* tachyzoites responsible for the induction of TNF-α in macrophages^46^,^47^,^48^. In our study, MIC3 was shown for the first time to be the relevant parasite molecule responsible for TNF-α induction (Fig. 7). MIC3 is known to be the adhesive component of the MIC3/8 complex^38^, and it has been proposed as a DNA vaccine candidate against toxoplasmosis^49^. These data highlight additional directions of study to understand the roles of MICs during infection. MIC3 not only is an exclusive protein in the invasion of *T. gondii* but also mediates the immunopathogenesis of toxoplasmosis. Hence, we imagine that MIC3 plays multiple roles during the entire course of toxoplasmosis. The MIC3-derived adhesin function may aid initial parasite invasion. Later, secreted MIC3 induces TNF-α secretion, reducing the proliferation of *T. gondii* tachyzoites; however, the accumulation of TNF-α also aggravated inflammation via T-cell-mediated tissue damage^36^.

MIC3 and other TNF-α-inducing molecules may have potential uses as immunomodulators. In contrast to LPS and total *T. gondii* ESAs, MIC3 triggers TNF-α synthesis without the potential side effects of simultaneous IFN-γ production (Fig. S5). TNF-α plays several therapeutic roles within the body, including immunostimulation, resistance to tumors, and sleep regulation^26^,^50^. TNF-α is also an important mediator of resistance against infectious diseases^22^,^51^,^52^,^53^,^54^,^55^. For example, in experimental leishmaniasis, insufficient TNF-α is associated with progressive disease and death^53^. In neosporiasis, exogenous TNF-α reduced *Neospora caninum* tachyzoites numbers in cultures in a dose-dependent manner^54^. In cerebral toxoplasmosis, TNF-α acts synergistically with IFN-γ, resulting in an antiparasitic mechanism in brain cells^22^.

However, other ESA proteins that were differentially expressed between strains RH and TgCtwh3 also played their roles because these proteins interact with each other, as shown by the correlation plot (Fig. 5). All differentially expressed ESA proteins should therefore be investigated as possible targets for the prevention of *T. gondii* infection, which causes serious health problems, including birth defects, worldwide. In addition to identifying MIC3 as a potent inducer of TNF-α production, this study also provides a vast resource of protein information specifically for the virulent, non-cystogenic *T. gondii* Chinese 1 TgCtwh3.

## Methods

### Preparation of ESAs from *T. gondii*

*T. gondii* RH and TgCtwh3 tachyzoites were preserved in liquid nitrogen and resuscitated in C57BL/6 mice by intraperitoneal inoculation. This study received approval from the Animal Ethics Committee of Nanjing Medical University, and all animal experiments were conducted in accordance with the approved guidelines (Permit Number: NJMU/IACUC 1403048). ESAs were prepared as described previously with some modifications^56^. Tachyzoites harvested from peritoneal fluid were filtered through a 3 μm pore size track-etched membrane (Shanghai Nengthink Filtration Technology Co. Ltd., Shanghai, China) to remove host cell debris. After filtration, more than 95% of the tachyzoites were confirmed to be viable using trypan blue (Gibco BRL, Gaithersburg, MD, USA). Then, 1 × 10^8^ tachyzoites were incubated in 10 ml of RPMI 1640 medium (Gibco BRL) under mild agitation for 3 h at 37 °C. The samples were then centrifuged at 1,000 × *g* for 10 min at 4 °C. The ESA-containing supernatant was diluted with PBS and concentrated to 2 ml using an Amicon Ultra-15 centrifugal filter unit (5000 MWCO, Millipore, Billerica, MA, USA). Endotoxin was then removed using AffinityPak Detoxi-Gel Endotoxin Removing Gel (Thermo, Fairlawn, OH, USA) and the ESA-containing fluid was filtered through a 0.22 μm filter (Millipore). Endotoxin levels in RH ESAs and TgCtwh3 ESAs were < 0.1 EU/mg protein, as determined by a Limulus assay (Xiamen Limulus Reagent Co. Ltd., Xiamen, China). The ESA samples were then treated with dithiothreitol as described in Supplementary Protocol 1. Two independent biological replicates were performed for each strain. Protein concentrations were assayed using the Bradford method.

### ESA protein digestion and iTRAQ labelling

The protein from the previous step was resuspended in digestion buffer (100 mM TEAB, 0.05% SDS) to a final concentration of 1 mg/ml. Equal aliquots (100 μg) from each sample were digested with trypsin (Sigma; 1:20 w/w added at 0 and 4 h) overnight at 37 °C and dried using a vacuum centrifuge.

From each *T. gondii* strain, two samples from separately propagated tachyzoites (biological replicates) were labelled using different isobaric tags (RH, 114- and 118-tags; TgCtwh3, 119- and 121-tags). The procedure is outlined in Supplementary Protocol 2 in the Supplementary Information.

### LC-MS/MS analysis

Each fraction was resuspended in buffer A (5% ACN, 0.1% FA) and centrifuged at 20, 000  × *g* for 10 min. The final concentration of peptide in the supernatant was approximately 0.5 μg/μl. LC-MS/MS was performed using a Triple TOF 5600 System (AB SCIEX, Framingham, MA, USA) coupled to an LC-20AD nanoHPLC (Shimadzu). The supernatant (5 μl) was loaded on the nanoHPLC by the autosampler onto a 2 cm C18 trap column. The samples were loaded at 8 μl/min for 4 min. The 60 min gradient was subsequently run at 300 nl/min, beginning with 5% buffer B (95% ACN, 0.1% FA) for 5 min, 5–35% buffer B for 35 min, followed by a 5-min linear gradient to 60%, a 2-min linear gradient to 80%, maintenance at 80% with buffer B for 2 min, and finally replacement with 5% Buffer B for 10 min. Data acquisition was performed using a Triple TOF 5600 System fitted with a Nanospray III source (AB SCIEX) and a pulled quartz tip as the emitter (New Objectives, Woburn, MA, USA). The procedure is outlined in Supplementary Protocol 3.

### Proteomic data analysis

The resulting MS/MS spectra were searched against the composite database of *T. gondii* GT1 and IPI_mouse available at ToxoDB^57^ and the European Bioinformatics Institute (EBI). The search parameters are presented in Supplementary Protocol 4.

Functional category analysis was performed using Blast2GO software (http://www.geneontology.org). The heat map was generated using MultiExperimentViewer (version 4.8.1) to visualize differences in protein expression^58^. The protein-protein correlation network was generated using Pearson’s correlation coefficient and visualized with Cytoscape software version 2.8.3^33^. The Pearson’s correlation coefficient for differentially expressed ESA proteins was calculated using R, and its significance was tested (*r* > 0.6 or < −0.6, *P* < 0.01).

### Real-time qRT-PCR

The mRNA levels in *T. gondii* or in mouse peritoneal macrophages were determined by qRT-PCR. RNA was extracted using TRIzol reagent (Invitrogen, San Diego, CA, USA). Genomic DNA was removed using DNase I (Thermo Fisher Scientific Fermentas, Vilnius, Lithuania) according to the manufacturer’s instructions. First-strand cDNA was synthesized from 4 μg total RNA using a RevertAid™ First Strand cDNA Synthesis Kit (Thermo Fisher Scientific Fermentas). Semi-quantitative RT-PCR was performed using SYBR Green PCR master mix (Applied Biosystems) and an ABI Prism 7300 sequence detection system (Applied Biosystems). Gene-specific primers were designed using Primer Premier 5.0, and the sequences of the primer pairs are listed in Supplementary Table S2. Changes in the fluorescence of SYBR Green I were monitored by the system software for every cycle. The threshold cycle and melting curves were measured automatically. Data are presented as the means ± S.D. from six independent measurements. GAPDH served as the internal control. The relative expression level of each gene was calculated using the 2^−(△△Ct)^ method.

### *In vivo* TNF-α production in mice after *T. gondii* Infection and quantification of *T. gondii* burden

*T. gondii* tachyzoites (1 × 10^6^) were intraperitoneally inoculated into groups of six-week-old C57BL/6 mice, which were purchased from the Model Animal Research Center of Nanjing University (Nanjing, China). The mice were sacrificed after 3 days. Serum levels of TNF-α were measured by ELISA according to the instructions of the manufacturer (eBioscience, San Diego, CA, USA). Spleens were harvested to assess the parasite burden. The procedure is outlined in Supplementary Protocol 5. All animal experiment protocols were approved by the Animal Ethics Committee of Nanjing Medical University. All experiments were performed in accordance with the approved guidelines.

### *In vitro* TNF-α production by mouse splenocytes in response to *T. gondii* tachyzoites

Splenocytes were harvested from six-week-old C57BL/6 mice. Next, RH or TgCtwh3 tachyzoites (1 × 10^6^) were inoculated into splenocytes (1 × 10^6^) *in vitro*. Supernatants were collected after 72 h and frozen at −80 °C for TNF-α determination.

### *In vitro* TNF-α production by mouse splenocytes or macrophages in response to *T. gondii* ESA or MIC3 stimulation

*T. gondii* ESAs or active *T. gondii* MIC3 protein (Abcam, Cambridge, UK) were treated with AffinityPak Detoxi-Gel Endotoxin Removing Gel (Thermo) to achieve an endotoxin level < 0.1 EU/mg. Splenocytes or peritoneal macrophages were harvested from six-week-old C57BL/6 mice. The ESAs or MIC3 protein were then used to stimulate splenocytes (2 × 10^6^) or peritoneal macrophages (1 × 10^6^) *in vitro*. Supernatants were collected after 72 h and frozen at −80 °C for TNF-α determination. Peritoneal macrophages were also harvested for qRT-PCR analysis. The morphology of peritoneal macrophages was analysed using a phase contrast microscope (Olympus, Tokyo, Japan). The procedure applied for the *in vitro* treatment of peritoneal macrophage is outlined in Supplementary Protocol 6.

All data are presented as the means ± S.D. from six independent experiments. Statistical tests to generate *P*-values are indicated in the corresponding figure legends.

### Concentration of MIC3 in *T. gondii* ESAs

ELISA was performed to quantitate MIC3 in *T. gondii* RH and TgCtwh3 ESAs. Serial dilutions of the active *T. gondii* MIC3 protein fragment were used as the standard. Ten micrograms of each ESA was immobilized and probed with mouse anti-*T. gondii* MIC3 antibodies (GeneTex, San Antonio, Texas, USA). Horseradish peroxidase (HRP)-labelled goat anti-mouse IgG (Santa Cruz Biotechnology, Santa Cruz, CA, USA) was used for detection. The plates were washed between each step to remove unbound antibodies. After the final wash, the plates were developed using tetramethylbenzidine and recorded using a Synergy HT Microplate Reader (BioTek, Winooski, VT, USA).

### Immunoprecipitation (IP) of MIC3 from ESAs

The IP of MIC3 from ESAs was performed as described previously with some modification^59^. For a single IP, 20 μl mouse monoclonal antibody to *T. gondii* MIC3 (anti-MIC3 mAb, GeneTex) was incubated with 200 μl protein G-agarose suspension (Sigma-Aldrich, St. Louis, MO, USA) at 4 °C for 3 h. Next, anti-MIC3 mAb conjugated to the protein G-agarose suspension was collected by centrifugation, mixed with ESAs (200 μg) and incubated with mixing at 4 °C for 3 h. Finally, the protein G-agarose was removed by centrifugation. The depletion of MIC3 from ESA was analysed by ELISA. Protein concentrations were assayed using the Bradford method. Equal amounts of ESAs and MIC3-depleted ESAs were loaded.

### Flow cytometry

To evaluate macrophage polarization, peritoneal macrophages from each mouse were stimulated with RH ESAs, TgCtwh3 ESAs, MIC3 or OVA in complete RPMI 1640 medium for 72 h at 37 °C in 5% CO_2_. After 72 h, the cells were collected and incubated with anti-CD16/32 blocking antibody (eBioscience) at 4 °C for 15 min. For purity analysis, the cells were incubated with PE-conjugated antibody against mouse F4/80 and PerCP-Cy5.5-conjugated antibody against mouse CD11b (BioLegend, San Diego, CA, USA). For M1 and M2 surface marker analysis, the cells were incubated with FITC-labelled anti-mouse CD16/32, APC-labelled anti-mouse CD206, and FITC- or APC- conjugated rat IgG2a isotype antibodies (all from BioLegend). The samples were then evaluated using a FACSVerse flow cytometer (BD Biosciences, San Jose, CA, USA) and analysed using FlowJo (Tree Star, Ashland, OR, USA).

## Additional Information

**How to cite this article**: Qiu, J. *et al*. Identification of a TNF-α inducer MIC3 originating from the microneme of non-cystogenic, virulent *Toxoplasma gondii. Sci. Rep.* **6**, 39407; doi: 10.1038/srep39407 (2016).

**Publisher's note:** Springer Nature remains neutral with regard to jurisdictional claims in published maps and institutional affiliations.

## Supplementary Material

Supplementary Information

## Figures and Tables

**Table 1:** *T. gondii* ESA proteins with different abundances in strains RH and TgCtwh3.

No.	ToxoDB ID^a^	Protein Name	Fold Changes (RH/TgCtwh3)^b^	Sequence Coverage (%)	Unique Peptides
1	TGGT1_019450	microneme protein MIC2	3.687	20.7	10
2	TGGT1_125360	MIC2-associated protein M2AP	4.323	10.0	3
3	TGGT1_121730	microneme protein MIC3	5.259	21.2	5
4	TGGT1_021130	microneme protein MIC4	2.774	19.3	10
5	TGGT1_029350	microneme protein MIC6	3.072	12.6	4
6	TGGT1_027570	microneme protein MIC9	3.014	11.0	7
7	TGGT1_102150	microneme protein MIC10	1.733	23.2	5
8	TGGT1_016150	microneme protein MIC11	1.687	21.6	4
9	TGGT1_060480	microneme protein MIC17A	9.806	16.8	4
10	TGGT1_062250	toxolysin TLN4	1.779	5.0	9
11	TGGT1_016650	subtilisin SUB1	5.344	8.2	4
12	TGGT1_085550	rhoptry protein ROP1	17.578	7.2	3
13	TGGT1_042710	rhoptry protein ROP5	2.496	8.9	4
14	TGGT1_075470	rhoptry protein ROP7	0.787	22.4	9
15	TGGT1_123520	rhoptry protein ROP15	1.334	21.6	6
16	TGGT1_063760	rhoptry protein ROP18	3.842	8.5	4
17	TGGT1_031600	rhoptry kinase family protein ROP40	1.994	20.0	9
18	TGGT1_041290	rhoptry neck protein RON8	2.904	8.5	22
19	TGGT1_082670	dense granule protein GRA3	5.811	10.4	2
20	TGGT1_102940	dense granule protein GRA9	0.614	18.9	5
21	TGGT1_034740	dense granule protein GRA12	1.360	22.7	9
22	TGGT1_121850	SAG-related sequence SRS17B	2.356	27.4	6
23	TGGT1_039470	SAG-related sequence SRS25	3.342	11.5	2
24	TGGT1_113990	SAG-related sequence SRS29C (SRS2)	1.359	12.6	4
25	TGGT1_072740	SAG-related sequence SRS35A	0.232	22.1	3
26	TGGT1_055870	SAG-related sequence SRS51 (SRS3)	6.591	8.3	4
27	TGGT1_092760	SAG-related sequence SRS52A	2.481	14.5	3

^a^ToxoDB release 8.0 (http://www.toxodb.org).

^b^Significant differences (*P* < 0.05), Student’s *t*-test.
