# Supplementary material for: Identification of a TNF-α inducer MIC3 originating from the microneme of non-cystogenic, virulent Toxoplasma gondii
Source: Sci Rep. 2016 Dec 21;6:39407. doi: 10.1038/srep39407 (PMC5175157; doi:10.1038/srep39407)
Supplement: Supplementary Information [file srep39407-s1.pdf]

Supplementary Information

Identification of a TNF-α inducer MIC3 originating from the microneme of non-cystogenic, virulent *Toxoplasma gondii*

Jingfan Qiu, Lijuan Wang, Rong Zhang, Ke Ge, Hongfei Guo, Xinjian Liu, Jinfeng Liu, Delong Kong, and Yong Wang*

Department of Pathogen Biology, Key Laboratory of Pathogen Biology of Jiangsu Province, Nanjing Medical University, Nanjing, 211166, China

14 pages, 6 protocols, 6 figures, 2 tables

* Correspondence

Yong WANG

Department of Pathogen Biology, Key Laboratory of Pathogen Biology of Jiangsu Province, Nanjing Medical University, No.101 Longmian Avenue, Nanjing, 211166, China

Email: yongwsh@njmu.edu.cn

Tel/Fax: +86 25 86869336

Supplementary Protocol 1

The ESA samples were treated for 1 h at 56°C with 10 mM DTT. Next, 55 mM iodoacetamide was added, and the samples were incubated for 45 min in the dark. Protein was precipitated by mixing the sample with ice-cold acetone (1:4, v/v) followed by incubation at -20°C for 2 h. After centrifugation for 20 min at 25,000×*g* (4°C), the precipitate was air-dried, resuspended in 0.5 M triethylammonium bicarbonate (TEAB) and sonicated on ice for 15 min. The samples were centrifuged at 25,000×*g* for 20 min at 4°C. The supernatant was then stored at −80°C until further use.

Supplementary Protocol 2

The iTRAQ labelling of peptide samples was performed using an iTRAQ reagent 8-plex kit (Applied Biosystems, Foster City, CA, USA). For each *T. gondii* strain, two samples from separately propagated tachyzoites (biological replicates) were labelled using different isobaric tags (RH, 114- and 118-tags; TgCtwh3, 119- and 121-tags). The samples were incubated for 2 h at room temperature and dried using a vacuum centrifuge.

The mixed iTRAQ-labelled peptides were dissolved in Buffer A (25 mM NaH_2PO_4 in 25% ACN, pH 2.7) and fractionated using an Ultremex SCX column (4.6×250 mm) with an LC-20AB HPLC Pump system (Shimadzu, Kyoto, Japan) at a flow rate of 1.0 ml/min. The 40 min HPLC gradient consisted of 5% buffer B (25 mM NaH_2PO_4, 1 M KCl in 25% ACN, pH 2.7) for 7 min, 5%-60% buffer B for 20 min, 60%-100% buffer B for 2 min, and 100% buffer B for 1 min, followed by 5% buffer B for 10 min. Elution was monitored by measuring the absorbance at 214 nm, and fractions were collected every 1

min. The eluted peptides were pooled into 20 fractions, desalted using a Strata X C18 column (Phenomenex, Torrance, CA, USA), and vacuum-dried.

Supplementary Protocol 3

Proteomic data acquisition was performed with a Triple TOF 5600 System. Data were acquired using an ion spray voltage of 2.5 kV, gas curtain of 30 psi, nebulizer gas of 15 psi, and an interface heater temperature of 150°C. The MS was operated with a resolution ratio of greater than or equal to 30,000 FWHM for TOF MS scans. Survey scans were acquired in 250 ms. A maximum of 30 product ion scans were collected if a threshold of 120 counts per second (counts/s) was exceeded and with a 2+ to 5+ charge state. The total cycle time was fixed at 3.3 s. The Q2 transmission window was 100 Da for 100%. Four time bins were summed for each scan at a pulser frequency value of 11 kHz by monitoring the 40 GHz multichannel TDC detector with four-anode channel ion detection. A sweeping collision energy setting of 35 ± 5 eV coupled with iTRAQ-adjusted rolling collision energy was applied to all precursor ions for collision-induced dissociation. Dynamic exclusion was set at 1/2 of the peak width (15 s), and then the precursor was refreshed from the exclusion list. The peak areas of the iTRAQ reporter ions reflected the relative abundance of the proteins in the samples. The triple TOF 5600 mass spectrometer for peptide identification has a mass accuracy of < 2 ppm.

Supplementary Protocol 4

The resulting MS/MS spectra were searched against the composite database of *T. gondii* GT1 (8100 sequences) and IPI_mouse (59534

sequences) available at ToxoDB (http://toxodb.org/common/downloads/release-8.0/TgondiiGT1/fasta/data/)[58] and the European Bioinformatics Institute (EBI) (ftp://ftp.ebi.ac.uk/pub/databases/IPI/last_release/current/) using Mascot software (version 2.3.02, Boston, MA). The search parameters were as follows: trypsin/P to allow fragments containing one missed cleavage; fragment mass spectrum (MS) tolerance, ± 0.1 Da; peptide MS tolerance, ± 0.05 Da; 100% carbamidomethylation of cysteine; variable conversion rate of N-terminal glutamine to pyroglutamic acid and methionine oxidation. All identified peptides had an ion score greater than the Mascot peptide identity threshold, and a protein was considered identified if at least one unique peptide match was available. For protein quantitation, at least two unique peptides, a ≥ 1.2-fold change, and $P < 0.05$ were required for significant change.

Supplementary Protocol 5

The *T. gondii* burden was quantitated as described previously with some modifications[1,2]. Splenic DNA was extracted using a QIAamp DNA Mini kit (Qiagen, Hilden, Germany) according to the manufacturer's instructions. DNA from each sample was analysed by qPCR. Parasite burden was expressed as the ratio of the *T. gondii* ITS1 gene (internal transcribed spacer 1) to the mouse glyceraldehyde 3-phosphate dehydrogenase (GAPDH) gene. The primers used are listed in Supplementary Table S2.

Supplementary Protocol 6

Peritoneal macrophages were isolated from C57BL/6 mice as described

previously[3]. Peritoneal macrophages were cultured in 6-well plates (Costar, Cambridge, MA, USA) at a density of 1 × 10^6 cells/well. For antigen treatment, macrophages were washed with PBS to remove non-adherent cells. Then 10 μg/ml RH ESAs, 10 μg/ml TgCtwh3 ESAs, 10 μg/ml OVA, MIC3 (1, 2, 4, 8 μg/ml), or OVA (1, 2, 4, 8 μg/ml) were added directly to each well with fresh medium, respectively. The cells were then incubated for 72 h at 37°C. Macrophages treated with OVA served as controls for M1 and M2 polarization.

References

1 Jauregui, L. H., Higgins, J., Zarlenga, D., Dubey, J. P. & Lunney, J. K. Development of a real-time PCR assay for detection of *Toxoplasma gondii* in pig and mouse tissues. *Journal of clinical microbiology* **39**, 2065-2071 (2001).

2 Ge, Y. Y. *et al.* Natural killer cell intrinsic toll-like receptor MyD88 signaling contributes to IL-12-dependent IFN-gamma production by mice during infection with *Toxoplasma gondii*. *International journal for parasitology* **44**, 475-484 (2014).

3 Zhu, J. *et al.* Parasitic antigens alter macrophage polarization during *Schistosoma japonicum* infection in mice. *Parasit Vectors* **7**, 122 (2014).

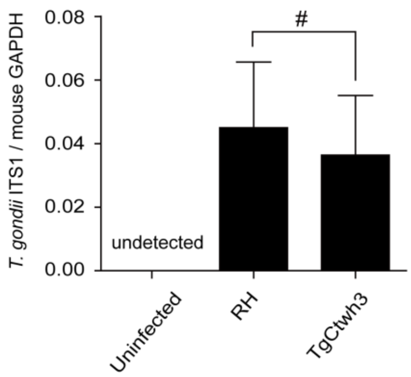

Supplementary Figure S1. Parasite burdens in the spleens of mice on day 3 p.i. with 10^6 RH or TgCtwh3 tachyzoites. The splenic parasite burdens were measured using real-time quantitative PCR and expressed as the ratio of the *T. gondii* internal transcribed spacer 1 (ITS1) gene to the mouse GAPDH gene. Data are represented as the means ± S.D. (n = 6). Significance was analysed using a two-tailed Student's *t*-test. # *P* > 0.05.

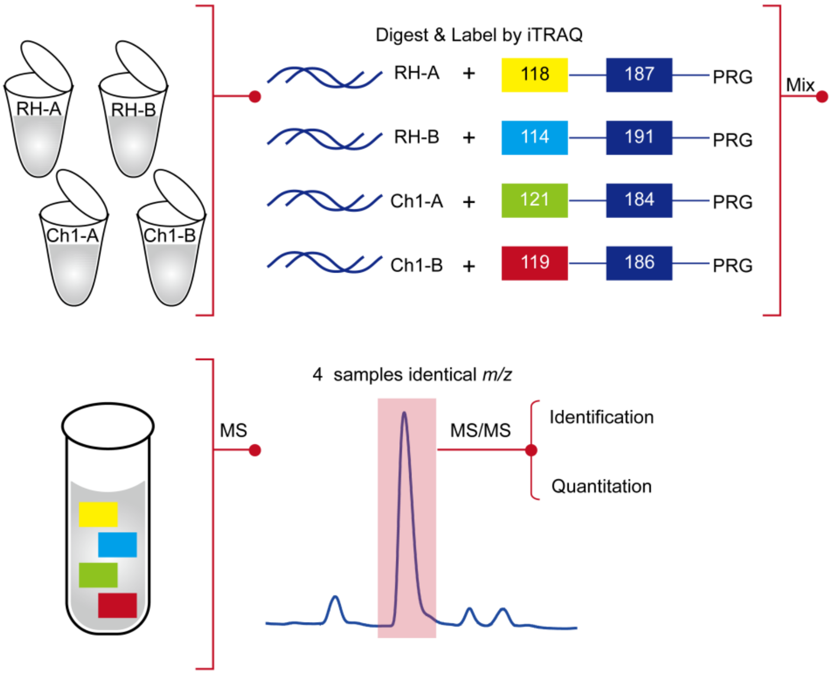

Supplementary Figure S2. Experimental process of the *T. gondii* ESA comparative proteomics study. Ch1-A and Ch1-B represent two biological replicates of the TgCtwh3 strain; RH-A and RH-B represent two biological replicates of the RH strain.

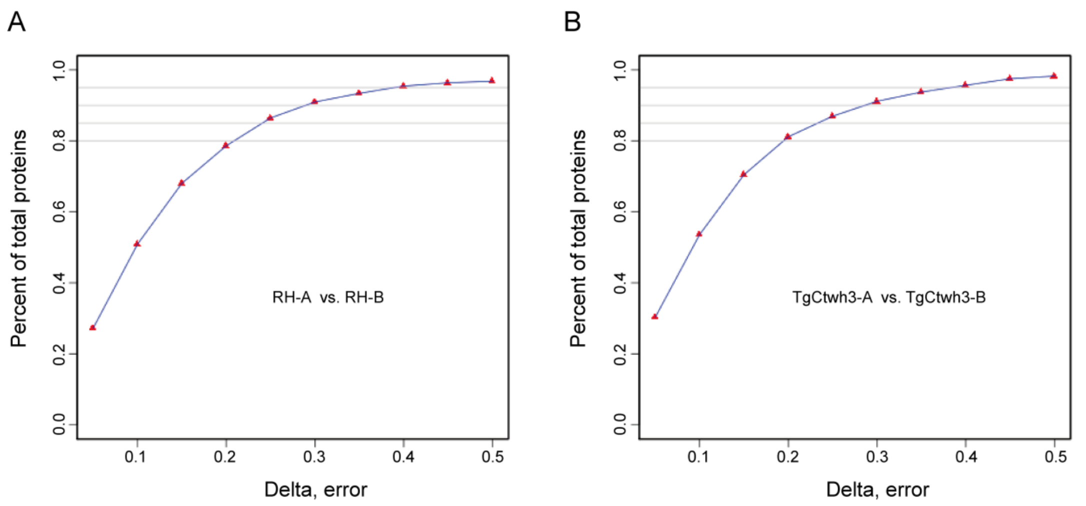

Supplementary Figure S3. Reproducibility of biological replicates. (A) RH strain, (B) TgCtwh3 strain.

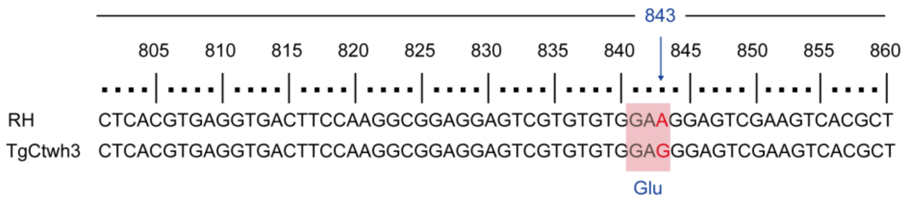

Supplementary Figure S4. Alignment of sequences of MIC3 from the *T. gondii* RH and TgCtwh3 strains. Three pairs of primers (Supplementary Table S2) were used to amplify the entire length of the MIC3 gene from *T. gondii* RH and TgCtwh3. We determined the nucleotide sequences of the MIC3 gene by sequencing. MIC3 nucleotide and amino acid sequences from the RH and TgCtwh3 strains were compared using SeqMan software. The nucleotide at position 843 is indicated by the square frame. The sequence of the MIC3 gene from the RH strain was identical to the sequence reported in GenBank (Accession: AF509564.1, GI: 20977535). Compared with the MIC3 gene from strain RH, the MIC3 gene from the TgCtwh3 strain contained a single

nucleotide mutation at 843 bp (A to G). However, the codons GAA and GAG both code for glutamic acid (E). This result confirmed that the amino acid sequences of MIC3 from *T. gondii* RH and TgCtwh3 were identical.

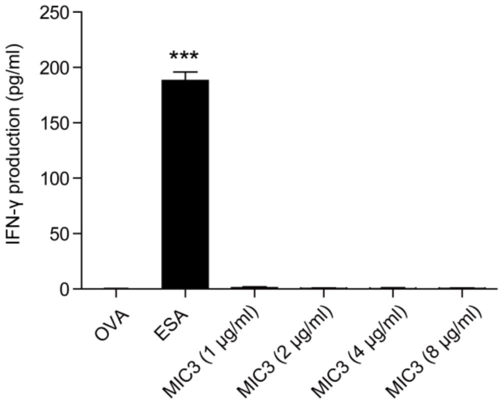

Supplementary Figure S5. Effects of MIC3 from *T. gondii* ESAs on IFN-γ secretion. Data are presented as the means ± S.D. (n = 6). Significance was analysed using one-way ANOVA. *** $P < 0.001$.

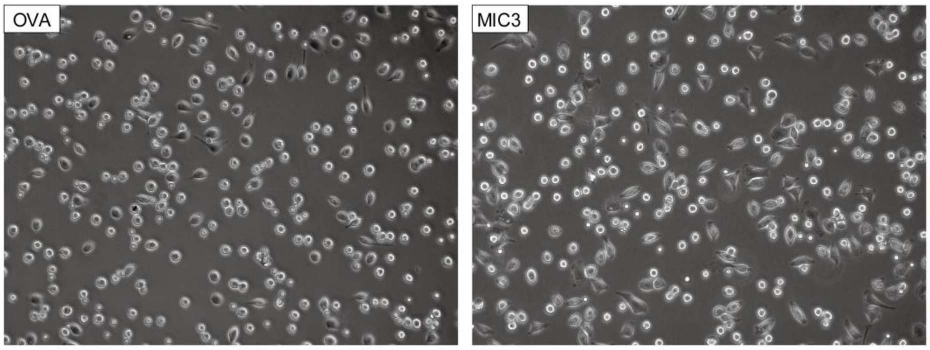

Supplementary Figure S6. Morphology of peritoneal macrophages treated with 1 μg/ml MIC3 or 1 μg/ml OVA for 48 h. Images were acquired using a phase contrast microscope (Olympus, Tokyo, Japan). Objective lens, ×20.

Supplementary Table S1. List of upregulated and downregulated *T. gondii* proteins identified and quantified by the iTRAQ analysis. [(a)] ToxoDB release 8.0 (http://www.toxodb.org). [(b)] Fold changes (RH vs. TgCtwh3) with significant differences ($P < 0.05$), Student's *t*-test.

No.	ToxoDB ID[(a)]	Description	Fold Changes (RH/TgCtwh3)[(b)]	Sequence Coverage (%)	Unique Peptides
1	TGGT1_008800	conserved hypothetical protein	2.429	11.7	3
2	TGGT1_016150	microneme protein MIC11	1.687	21.6	4
3	TGGT1_016570	membrane-attack complex /perforin domain-containing protein	4.259	9.6	6
4	TGGT1_016650	subtilisin SUB1	5.344	8.2	4
5	TGGT1_018190	hypothetical protein	1.318	13.9	5
6	TGGT1_019450	microneme protein MIC2	3.687	20.7	10
7	TGGT1_021130	microneme protein MIC4	2.774	19.3	10
8	TGGT1_021470	hypothetical protein	1.738	15.0	3
9	TGGT1_024250	hypothetical protein	1.623	14.0	6
10	TGGT1_025360	heat shock protein, putative	2.356	42.3	17
11	TGGT1_027570	microneme protein MIC9	3.014	11.0	7
12	TGGT1_029350	microneme protein MIC6	3.072	12.6	4
13	TGGT1_031600	rhoptry kinase family protein ROP40	1.994	20.0	9
14	TGGT1_032990	macrophage migration inhibitory factor, putative	1.468	20.7	2
15	TGGT1_033690	conserved hypothetical protein	1.843	2.5	2
16	TGGT1_034740	dense granule protein GRA12	1.360	22.7	9
17	TGGT1_035530	conserved hypothetical protein	3.130	14.0	4
18	TGGT1_039470	SAG-related sequence SRS25	3.342	11.5	2

19	TGGT1_041290	rhoptry neck protein RON8	2.904	8.5	22
20	TGGT1_042710	rhoptry protein ROP5	2.496	8.9	4
21	TGGT1_051660	hypothetical protein	1.940	9.3	2
22	TGGT1_055870	SAG-related sequence SRS51 (SRS3)	6.591	8.3	4
23	TGGT1_058380	conserved hypothetical protein	1.528	34.1	4
24	TGGT1_060480	microneme protein MIC17A	9.806	16.8	4
25	TGGT1_062250	toxolysin TLN4 / metalloprotease	1.779	5.0	9
26	TGGT1_063760	rhoptry protein ROP18 / protein kinase domain-containing protein	3.842	8.5	4
27	TGGT1_064690	conserved hypothetical protein	2.268	9.6	10
28	TGGT1_066110	conserved hypothetical protein	1.761	1.7	2
29	TGGT1_071510	conserved hypothetical protein	6.784	30.1	10
30	TGGT1_074260	conserved hypothetical protein	1.500	48.1	5
31	TGGT1_081090	conserved hypothetical protein	3.957	13.3	3
32	TGGT1_082100	heat shock protein 70kD, putative	1.747	9.9	8
33	TGGT1_082670	dense granule protein GRA3	5.811	10.4	2
34	TGGT1_085550	rhoptry protein ROP1	17.578	7.2	3
35	TGGT1_086210	conserved hypothetical protein	1.450	24.1	5
36	TGGT1_092760	SAG-related sequence SRS52A	2.481	14.5	3
37	TGGT1_100270	conserved hypothetical protein	1.428	4.3	4
38	TGGT1_102150	microneme protein MIC10	1.733	23.2	5
39	TGGT1_113990	SAG-related sequence SRS29C (SRS2)	1.359	12.6	4
40	TGGT1_115220	conserved hypothetical protein	2.473	19.5	3
41	TGGT1_115370	conserved hypothetical protein	1.479	22.0	6
42	TGGT1_117730	conserved hypothetical protein	5.576	35.2	3

43	TGGT1_121730	microneme protein MIC3	5.259	21.2	5
44	TGGT1_121850	SAG-related sequence SRS17B	2.356	27.4	6
45	TGGT1_123520	rhoptry protein ROP15	1.334	21.6	6
46	TGGT1_123900	conserved hypothetical protein	2.388	58.1	9
47	TGGT1_125220	conserved hypothetical protein	1.951	12.7	4
48	TGGT1_125360	MIC2-associated protein M2AP	4.323	10.0	3
49	TGGT1_004090	conserved hypothetical protein	0.425	24.8	3
50	TGGT1_029790	conserved hypothetical protein	0.313	18.2	3
51	TGGT1_032760	hypothetical protein	0.667	16.7	14
52	TGGT1_053770	conserved hypothetical protein	0.424	12.8	4
53	TGGT1_057870	conserved hypothetical protein	0.719	2.7	4
54	TGGT1_072740	SAG-related sequence SRS35A	0.232	22.1	3
55	TGGT1_075470	rhoptry protein ROP7	0.787	22.4	9
56	TGGT1_092700	conserved hypothetical protein	0.627	9.5	6
57	TGGT1_096800	conserved hypothetical protein	0.175	19.4	2
58	TGGT1_098250	receptor for activated C kinase, RACK protein, putative	0.642	41.4	10
59	TGGT1_098340	ABC transporter, putative	0.621	10.8	5
60	TGGT1_098470	KH domain-containing protein, putative	0.782	14.3	10
61	TGGT1_102220	conserved hypothetical protein	0.800	37.9	11
62	TGGT1_102940	dense granule protein GRA9	0.614	18.9	5
63	TGGT1_112260	cell division protein, putative	0.754	25.3	16
64	TGGT1_115180	NBP2B protein, putative	0.489	7.9	4
65	TGGT1_118190	membrane-associated calcum-binding protein, putative	0.619	24.8	6
66	TGGT1_125960	hypothetical protein	0.646	9.8	5

Supplementary Table S2. Primers used in this study.

Name	Sequence (5'-3')	Use
GAPDH-F	CTCATTGTCGGCGGAAAGG	Real-time RT-PCR amplification of *T. gondii GAPDH*
GAPDH-R	GGTGGACTCGCAGATGTAGTGG	
085550-F	CAGGGACCAGTTCGTCAAAA	Real-time RT-PCR amplification of *ROP1*
085550-R	GAAACCACTCCTCACTCCACC	
082670-F	GATTCTCGCCGCCTACTACA	Real-time RT-PCR amplification of *GRA3*
082670-R	CAGCACCCTCATCCTTTGG	
121730-F	AGTCACGCTGGCTGAGAAATG	Real-time RT-PCR amplification of *MIC3*
121730-R	GGAGGTTGCGGAAGCAGAT	
063760-F	AGGAAAGGGCTCAACACCG	Real-time RT-PCR amplification of *ROP18*
063760-R	GGAACCCGAGACTTCACCATC	
029350-F	TCGACGCCAGTGCCCATAT	Real-time RT-PCR amplification of *MIC6*
029350-R	CGACTTTCACCTTCATCTTCCCT	
GAPDH-F	AGGTCGGTGTGAACGGATTTG	Real-time RT-PCR amplification of mouse *GAPDH*
GAPDH-R	TGTAGACCATGTAGTTGAGGTCA	

IL-6-F	GAGGATACCACTCCCAACAGACC	Real-time RT-PCR amplification of *IL-6*
IL-6-R	AAGTGCATCATCGTTGTTCATACA	
iNOS-F	GGAGCGAGTTGTGGATTGTC	Real-time RT-PCR amplification of *iNOS*
iNOS-R	GTGAGGGCTTGGCTGAGTGAG	
Arg-1-F	CAGAAGAATGGAAGAGTCAG	Real-time RT-PCR amplification of *Arg-1*
Arg-1-R	CAGATATGCAGGGAGTCACC	
IL-10-F	GACCAGCTGGACAACATACTGCTAA	Real-time RT-PCR amplification of *IL-10*
IL-10-R	GATAAGGCTTGGCAACCCAAGTAA	
Fizz1-F	TCCCAGTGAATACTGATGAGA	Real-time RT-PCR amplification of *Fizz-1*
Fizz1-R	CCACTCTGGATCTCCCAAGA	
Toxo ITS1-F2	CAAGAAGCGTGATAGTATCG	Real-time PCR amplification of *ITS1*
Toxo ITS1-R2	CTGAAGAAACTCCTGGAAATC	
MIC3-Q1	GTCACTCAACTTGCTGCTA	PCR amplification of a part of the MIC3 gene
MIC3-Q2	CGAGGCGTCATCAATACAT	
121730-NC1	AAACATATGCGAGGCGGGACGTCCGCGCTG	PCR amplification of a part of the MIC3 gene
121730-NC2	AAAAAGCTTCTGCTTAATTTTCTCACACGT	
MIC3-W1	GCAGTTCGCTCATCTACC	PCR amplification of a part of the MIC3 gene

MIC3-W2	TCTCAATCCTTGGCTTAGTG	
